# ShinyEvents: harmonizing longitudinal data for real world survival estimation

**DOI:** 10.21203/rs.3.rs-7231850/v1

**Published:** 2025-08-06

**Authors:** Alyssa Obermayer, Joshua Davis, Divya Priyanka Talada, Mingxiang Teng, Steven Eschrich, Vivien Yin, Daniel Spakowicz, Dipankor Dhrubo, Robert J Rounbehler, Michelle L. Churchman, Ahmad A. Tarhini, Xuefeng Wang, Sumati Gupta, Joseph Markowitz, Jeremy Goecks, Roger Li, Rodrigo Rodriguez-Pessoa, Brandon J. Manley, Aik-Choon Tan, G Daniel Grass, Dung-tsa Chen, Timothy I. Shaw

**Affiliations:** H. Lee Moffitt Cancer Center and Research Institute; H. Lee Moffitt Cancer Center and Research Institute; H. Lee Moffitt Cancer Center and Research Institute; H. Lee Moffitt Cancer Center and Research Institute; H. Lee Moffitt Cancer Center and Research Institute; H. Lee Moffitt Cancer Center and Research Institute; The Ohio State University Comprehensive Cancer Center; The Ohio State University Comprehensive Cancer Center; Aster Insights; Aster Insights; H. Lee Moffitt Cancer Center and Research Institute; H. Lee Moffitt Cancer Center and Research Institute; Huntsman Cancer Institute; H. Lee Moffitt Cancer Center and Research Institute; H. Lee Moffitt Cancer Center and Research Institute; H. Lee Moffitt Cancer Center and Research Institute; H. Lee Moffitt Cancer Center and Research Institute; H. Lee Moffitt Cancer Center and Research Institute; Huntsman Cancer Institute; H. Lee Moffitt Cancer Center and Research Institute; H. Lee Moffitt Cancer Center and Research Institute; H. Lee Moffitt Cancer Center and Research Institute

## Abstract

Longitudinal data analysis of the patient’s treatment course is critical to uncovering variables that influence outcomes. However, existing tools have significant limitations in integrating multilayered time-series data. Here, we developed ShinyEvents, a web-based framework for complex longitudinal data analysis. ShinyEvents allows users to upload data and generate interactive timelines of the patient’s clinical events. Our tool can perform cohort-level analysis, including the assignment of treatment clusters and clinical endpoints. Our tool also provides informative cohort visualizations, such as a Sankey diagram of the treatment line and Swimmer diagram of the clinical course. Finally, our tool can infer a real-world progression-free survival (rwPFS) based on user-defined endpoints to perform Kaplan-Meier and Cox proportional hazards regression analysis. With these features, the tool can then associate the lines of treatment with clinical outcomes. Altogether, ShinyEvents facilitates the integration of multilayered longitudinal data and enables survival analysis in real-time. A live link to the tool is available https://shawlab-moffitt.shinyapps.io/shinyevents/.

## Introduction

Longitudinal real-world data (RWD) are complex and heterogeneous, with inconsistent quality that makes them difficult to utilize for several reasons^1^. First, RWD is observational and often unstructured, varying in naming due to different documentation practices across providers^2,3^. Second, RWD can be challenging to analyze due to their overwhelming size, as they are generated from dynamic datasets from private and public repositories, such as the Oncology Research Information Exchange Network (ORIEN) AVATAR^4–8^, FLATIRON^9^, and AACR Project GENIE^10^. Third, RWD may lack important annotations for research purposes, such as treatment response labeling or surrogate endpoints^1^. Therefore, establishing standardized approaches for visualizing longitudinal data will be essential to leverage RWD to inform best practices in clinical decision-making and for the planning of future clinical trials.

Several tools have been developed for visualizing longitudinal clinical data, including cBioPortal^11^, OncoThreads^12^, Read-TV^13^, PlotTwist^14^, and Pergola-web^15^. These tools can be categorized based on the following objectives: 1) visualization of outcome measurements over time, 2) the sequence of the treatment course relative to the genomics data, and 3) the identification of temporal patterns. While these longitudinal visualization methods are useful in their respective field, they were primarily designed with a single focus, such as genomic profiling or blood/serum measurements. Notably, there is no existing tool for cohort-level analysis of treatment-associated information from RWD, especially tools that can define treatment response and surragate endpoint for survival analysis, which is critical for disease trajectory modeling^16,17^ and personalized decision support^18^.

**Figure 1:**
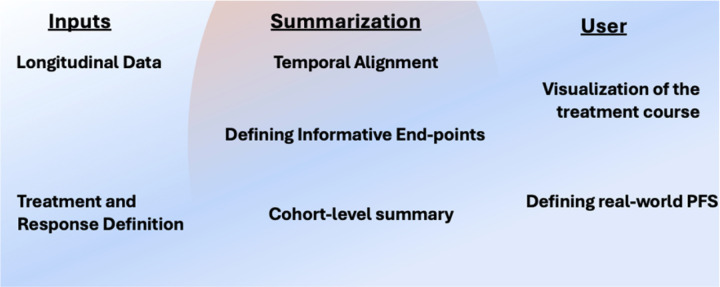
ShinyEvents integrates longitudinal data as input. The tool then aligns and summarizes the events into clusters. The tool provides effective visualization while allowing the user to define informative real-world PFS, which can then be associated with other biomarkers generated within the study.

To address these challenges, we developed a web-based viewer, ShinyEvents, that facilitates the analysis and presentation of longitudinal data focusing on the length of treatment, clinical response, and overall survival (Fig. 1). In addition to the time-series visualization, the tool can cluster treatment-associated data into treatment lines, define endpoints for real-world progression-free survival (rwPFS), and summarize population characteristics. We applied a public GENIE non-small cell lung cancer (NSCLC) cohort as well a dataset of patients with non-metastatic bladder cancer treated by cystectomy at Moffitt Cancer Center to demonstrate the functionalities of ShinyEvents.

## Results

### ShinyEvents Preprocessing with the GENIE non-small cell lung cancer cohort example

**Figure 2:**
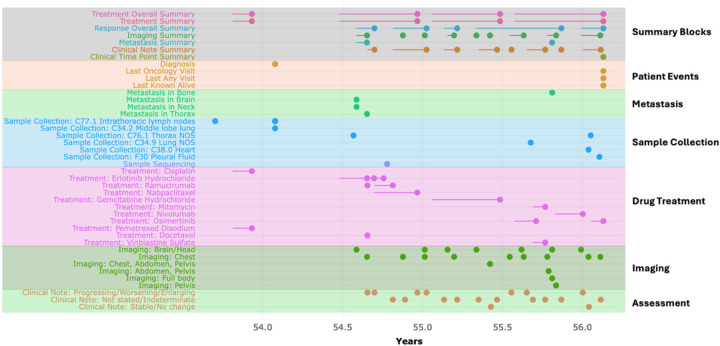
Patient-level summary of a patient with lung adenocarcinoma. Each block of longitudinal data is highlighted with a different color. The “Summary Block” provides a high-level summary of each set of longitudinal data.

As an example, we assembled a population of non-metastatic (stage II and III) NSCLC patients with adenocarcinoma histology from GENIE^19^. We collected data associated with the patient demographics, pathological staging, gene panel sequencing, treatment, imaging, and clinical notes. We filtered the cohort based on diagnosis stage II and III and restricted it to adenocarcinoma patients treated with Cisplatin/Vinorelbine or Cisplatin/Pemetrexed (n = 71). During the preprocessing step, the ShinyEvent would guide the user in generating an Events table indexed based on the patient’s age and their unique identifier. Furthermore, the following characteristics were defined for each event: event name (timeseries name), treatment (oncologic medicine, surgery, and radiation), response (potential end point), and duration of the event (start and end time points). For each patient, the tool would generate a Swimmer's plot to visualize the multi-dimensional data available for that patient. An example of a patient with an extensive history of adenocarcinoma lung cancer is presented in Fig. 2, with each category of longitudinal data harmonized across the timeline. Key Preprocessing steps are documented in the supplementary method and tutorial page in https://shawlab-moffitt.shinyapps.io/shinyevents/.

### Navigating in ShinyEvents

ShinyEvents offers four categories of analysis. 1) Patient Visual Analytics, 2) Treatment-associated Analytics, 3) Time-to-Event Analysis, and 4) Cohort Overview. Two key files are required as initial input. 1) a tab-separated text or Excel file with longitudinal event information. 2) a parameter file defining the Data table, Event Name, Category, Start Time, and End Time. The user can assign events as Treatment, Pathology, Clinical Response, and Imaging Assessment, which will facilitate the downstream analytical workflow. In general, the tool is organized with user input selection on the left panel of the graphical user interface, while the right panel displays the visual output, accompanied by a downloadable table below. When setting up an institutional local instance of ShinyEvents, the tool can be password protected if personal health information is included. A live version of the tool is accessible from https://shawlab-moffitt.shinyapps.io/shinyevents/. The Shiny app does not save or retain user uploaded files. A complete list of features is described in Table 1 and below.

**Figure 3:**
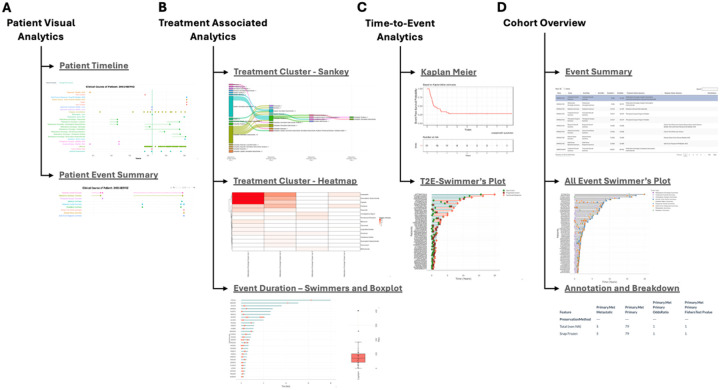
ShinyEvents Tab-based Navigation. **A)** Patient Visual Analytics. **B)** Treatment Associated Analytics. **C)** Time-to-Event Analytics. **D)** Cohort Overview.

**Patient Visual Analytics** offers a patient-centric view of longitudinal data. The user can perform generalized filtering of the patient population. Major clinical events, such as metastasis and progressive disease, can be highlighted in the visualization. Based on the filtered patient population, the user can explore the patient’s events throughout the treatment course with mouse-over functionality to highlight additional details of an event. As cancer care often involves a multimodal regimen encompassing systemic therapies, surgical procedures, and radiation, representing these interventions as an aggregated event facilitates computational modeling and downstream analytical workflows. Thus, we implemented an approach to perform clustering of temporally proximal events within a user-defined timeframe (i.e., one month window). The aggregated events can then be visualized in the Patient Event Summary tab (Fig. 3A).

**Figure 4:**
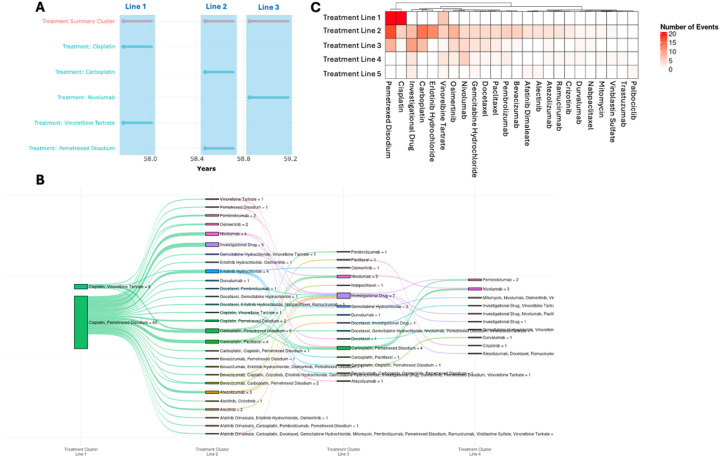
ShinyEvents facilitates the exploration of the treatment data. **A)** The treatment for each patient is grouped into Treatment Summary Cluster, defining the treatment lines. **B)** The user can define treatment clusters and visualized as a Sankey plot of the treatment line. **C)** The heatmap shows the number of patients treated with the drug in each treatment line. Treatment cluster based on a 3 month window in the GENIE NSCLC cohort.

**Treatment-associated Analytics** provides the user the ability to aggregate treatments into lines-of-treatment (Fig. 3B). Each treatment within a specified window is grouped together as individual treatment lines, which are named the **Treatment Summary Cluster** (Fig. 4A). A Sankey plot can then be used to visualize the lines of treatment across the cohort (Fig. 4B), where the width of the bars and lines represent the number of patients in that group and their treatment path. For example, in GENIE, patients with lung adenocarcinoma were first treated with Cisplatin/Pemetrexed or Cisplatin/Vinorelbine followed by diverse second line treatments which included EGFR-inhibitors (e.g., Erlotinib, Osimertinib, Afatinib), ALK-inhibitors (Alectinib/Crizotinib), Bevacizumab (anti-VEGF-antibody), Carboplatin, and immune checkpoint inhibitors (e.g., Pembrolizumab, Nivolumab, Durvalumab, Atezolizumab, and Ipilimumab). This can then be further visualized using a Heatmap (Fig. 4C), which illustrates the frequency of a particular treatment across treatment lines and individual patients (Supplementary S1). Overall, the Sankey plot and heatmap shows primary treatments with Cisplatin/Pemetrexed or Cisplatin/Vinorelbine followed by next line treatment with either 1) an investigational agent (clinical trial) if available, 2) salvage therapy with next line chemotherapy (carboplatin), 3) profiling with EGFRmut or ALK-fusion to give targeted agents, 4) immunotherapy agents, 5) and CDK4/6 inhibitor (palbociclib) after multiple lines of unsuccessful therapy. The duration of the treatment can be evaluated by a Swimmer’s plot, Box plot, and Heatmap to identify patients with a durable treatment. Altogether, the analytics can group patients with similar treatment regimens for downstream analysis.

**Time-to-Event Analysis** enables users to perform survival analysis (Fig. 3C). To perform survival analysis, the tool enables the user to define start and end points based on the patient’s timeline. An overview of the endpoint definition categories is presented in Table 2. Typically, the starting point is based on an oncologic treatment, treatment line, diagnosis, and molecular profiling of a subgroup of patients with a similar clinical baseline. The combination of endpoints would then dictate the type of clinical endpoint analysis. To estimate overall survival, we would use death as an endpoint. To estimate time to treatment change, we would use the next treatment line as an endpoint. To estimate a real-world progression-free survival (rwPFS), we would use the following four endpoints as progression events, including pathological recurrence, death, and radiological progression/metastasis. The output of these estimations can then be utilized for downstream survival analysis^20^. Altogether, ShinyEvents facilitates the estimation of rwPFS from the longitudinal data.

**Figure 5:**
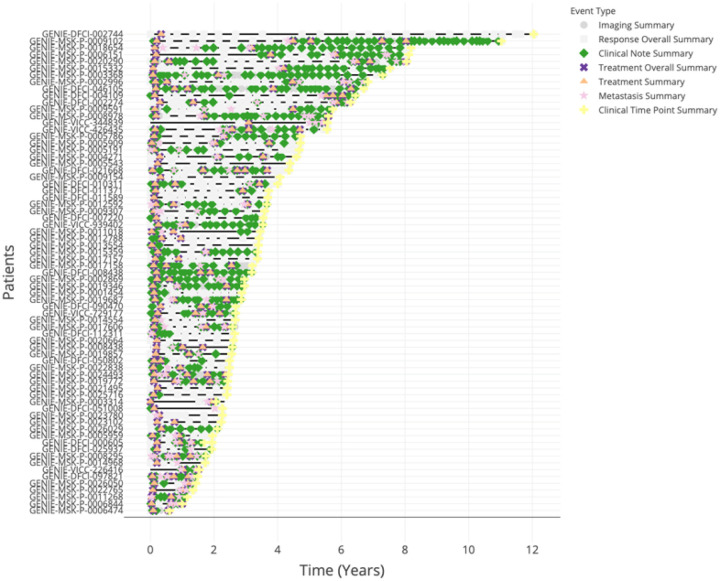
ShinyEvents facilitates the generation of a Swimmer’s Plot summary. Each node shows an event summary.

**Cohort Overview** ShinyEvents provides several methods for summarizing cohort-level information (Fig. 3D). Based on the GENIE lung adenocarcinoma population, the clinical table’s data elements can be viewed through an expanded tree viewer (Supplementary Figure S2A). The user can subset the data and generate a tabular summary of molecular and patient/sample data (Supplementary Figure S2B). A Swimmers plot can then be used to highlight the clinical course of each patient as well as cross cohort heterogeneity (Fig. 5). From the Swimmer’s plot, the user can cursor over (mouse over) each event for a dropdown with additional details, such as location of treatment site. The tool can stratify patients based on clinicopathologic characteristics, such as sex, clinical grade, and tumor histology. Furthermore, the user can position the major event (such as medication) relative to another event (i.e., sequencing data), annotating the molecular profiling before or after a particular drug regimen for downstream sequencing analysis. Altogether, this collection of tools offers several important preprocessing step overviews.

A live version of the GENIE data is available https://shawlab-moffitt.shinyapps.io/aacr_genie_nsclc_squamouscell_shinyevents/, and a tutorial of the tools is also made available through our GitHub page https://github.com/shawlab-moffitt/shinyEvents.

### Use case. Comparing real-world progression-free survival between line-of-treatment

For our separate use case example, we assembled a population of patients diagnosed with non-metastatic muscle-invasive bladder cancer treated with neoadjuvant chemotherapy followed by surgical resection at Moffitt Cancer Center who were also enrolled in the Oncology Research Information Exchange Network (ORIEN) AVATAR project. We collected tables associated with diagnosis, outcome, vital status, metastatic disease, and treatment. Altogether, the cohort consisted of 51 patients with a median age of 64 (range: 45–82) years at diagnosis.

**Figure 6:**
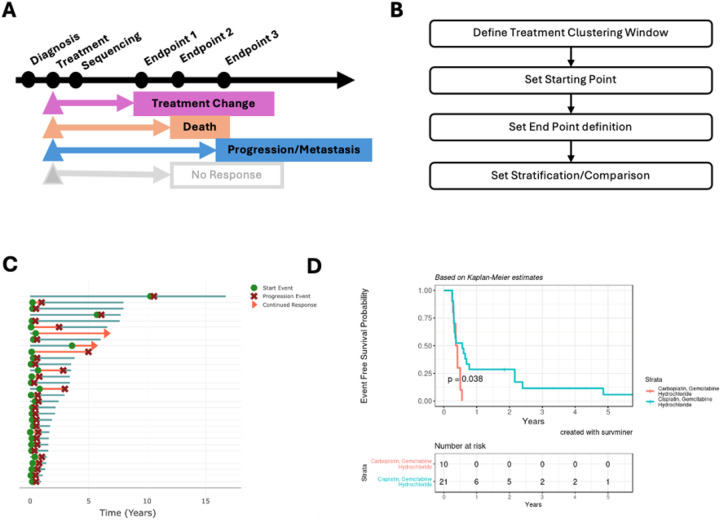
Defining informative endpoints. **A)** Endpoint definition that can be defined. **B)**Four key decisions required in the user interface. These include 1. setting the treatment clustering window, 2. selecting the starting time, 3. defining the surrogate end point, and 4. defining the groups for the survival analysis. **C)** Swimmer plot example showing starting neo-adjuvant treatment with Cisplatin/Gemcitabine or Carboplatin/Gemcitabine in patients with bladder cancer; the endpoint defined was based on treatment change, death, progression, and metastasis. **D)**The real-world progression-free survival of patients with bladder cancer was stratified based on treatment with carboplatin- or cisplatin-based treatment.

We have implemented a general strategy to define treatment-associated rwPFS. We first summarized the treatments rendered into treatment lines, revealing that the patients are predominantly treated with neoadjuvant Gemcitabine, and Cisplatin/or Carboplatin as first-line therapy (Supplementary Figure S3A). Using a Sankey plot, we found that line 1 primarily consisted of the Carboplatin Gemcitabine combination (n = 10) and the Cisplatin Gemcitabine combination (n = 21) (Supplementary Figure S3A, B). To estimate an rwPFS, we defined progression end points to be associated with treatment change, progression/metastasis, and death (Fig. 6A). To perform this analysis, the user would need to perform the following steps (Fig. 6B: 1) define a window for clustering the samples (Supplementary Figure S4A), 2) select a starting time point based on a specific treatment (Supplementary Figure S4B), 3) defining the surrogate endpoint (Supplementary Figure S4C), and 4) define the two groups for survival analysis (Supplementary Figure S4D). A tutorial video is presented in the tutorial section, **Time-To-Event Analysis**. A Swimmer’s plot was used to verify the start and end point of the patient’s clinical course (Fig. 6C). We performed a Kaplan-Meier analysis and found that patients treated with Carboplatin/Gemcitabine tend to have a worse clinical outcome compared to those treated with the Cisplatin/Gemcitabine combination (Fig. 6D). Clinically, Carboplatin may be given to patients with poor renal function or performance status, potentially explaining this observation.

## Discussion

The visualization of longitudinal data improves our understanding of the clinical course encompassing various lines of treatment rendered^21^. Current longitudinal data analytics primarily focuses on a single modality that is genomic-centric, which is often limited to a single biopsy or surgery throughout the treatment course^22^. Notably, there is a lack of visualization tools that can facilitate time-to-event analysis. Here we developed a ShinyEvents framework, that offers an expanded function for the user to define clinical start and endpoint for real-world survival analysis. Our use case highlights our tool’s ability to evaluate treatment regimen and its association with progression-free survival in a real-world bladder cancer cohort from Moffitt Cancer Center. Our tool can also be easily adapted to other publicly available longitudinal datasets, such as GENIE and Flatiron. We anticipate that this tool will be valuable to other institutions, allowing them to effectively utilize their private institutional patient treatment data for meaningful analysis and to derive clinical insights.

As the field moves toward developing personalized models grounded in RWD, effective preprocessing of time-series data and extraction of clinically relevant features will be essential to realizing the potential of the digital twin technology^23^. To accomplish this goal, it will be critical to maintain effective interaction between clinician, bioinformatician, biostatistician, and machine learning experts, as each brings a unique expertise that maximizes the utility of the patient’s longitudinal clinical information^24–26^. One of the major goals of ShinyEvents is to provide web visualization for effective interdisciplinary communication. The integration of clinical knowledge from experienced clinicians is critical to the development of a valid digital twin model, ensuring that the model captures the complexity of patient care and aligns with real-world clinical practice. Furthermore, there is the need to ensure robustness of the applied statistics and genomic inquiries that are required, which is often dependent on the chosen clinical end point. As the field advances toward big data–driven science, it is crucial to avoid indiscriminate data input, especially in machine learning applications where the risk of selection bias is pronounced. In summary, ShinyEvents fulfills a critical unmet need of a web-based tool that facilitates joint interaction between clinicians and data scientists to enable the development of robust and clinically meaningful models.

One major limitation of our tool is that it is not optimized for changepoint analysis from longitudinal measurements without extensive preprocessing. In its current version, the tool is designed for case-by-case evaluation of a patient’s disease; however, while it is theoretically possible, a systematic algorithm to determine disease stage (such as early non-metastatic, locally advanced, and metastatic) over the course of their disease can be established. We aim to incorporate these features in future versions of the tool.

## Methods

### Sofware Implemention R Shiny and environmental setup.

The ShinyEvents R Shiny application and package was built using R version 4.4.1 and can be installed via GitHub. To setup a ShinyEvents application interface a parameter file is required. This file outlines the data tables, event column names, and event descriptions which guide the app during startup to generate a comprehensive event data table which fuels the applications functionality. A detailed explanation of how to set up this parameter file can be found in the Github Page https://github.com/shawlab-moffitt/shinyEvents. To deploy the application the app folder should be structured to include the ‘app.R’ script, the ‘R’ folder, the parameter file, and the supplementary files that are annotated in the parameter file. At the top of the ‘app.R’ script there is a line to designate the file name and path of the parameter file “Event_Param_File” and if previously processed, the user can define the event data file and patient annotation file. With these criteria met, the user can deploy the app via the ‘Run App’ button or using the ‘runApp’ R function.

### Online User Tutorial Page

Within the ShinyEvents application users can view a tutorial page with an in-depth guide on file formatting as well as video tutorials and helpful text that overview the user interface. In the guide on file formatting, we breakdown the input file options to further explain what data is required at minimum and provide descriptions and examples for the file contents (Supplementary Figure S5A, S5B). This will cover the event data file and optional supplementary data that can be uploaded directly through the application interface, including a figure outlining how these files can be connected in the app. For more advanced use, we demonstrate how to set up the applications working directory and assemble a parameter file that can be read in through the ‘app.R’ script which guides the generation of detailed event data for exploration in the app. To assist users, we provide an R function that can be used as a pre-processing step to derive the event data from the parameter file. This is recommended when deploying the app with a parameter file or working with larger data sets, as the application startup may require additional computing time if the event data needs to be generated. Additionally, we include tutorial videos and helpful tips that demonstrate the features available in the app.

### Online Example

Source code and tutorial to ShinyEvents can be accessed on our GitHub page https://github.com/shawlab-moffitt/shinyEvents which also contains example data, instructions on setting up a local environment for the application, and R functions to assist in data pre-processing. A version of the tool is accessible by https://shawlab-moffitt.shinyapps.io/shinyevents/. Here users can load example data provided in the app to view the appropriate formatting and explore the features available. Within the app we provide a tutorial page, helpful tips, as well as a guided layout to simplify data upload and app customization. A Zenodo DOI is available https://doi.org/10.5281/zenodo.16527381.

### Quantification and Statistical Analysis

Highlight details about the Kaplan Meier analysis, Fisher’s Exact Test. Statistical tests were based on functions implemented in R.

## Supplementary Files

This is a list of supplementary files associated with this preprint. Click to download.

- ShinyEventsSupplementaryFiguresv120250724.pdf

## Figures and Tables

**Table 1:** List of Features.

	Feature	Description
**Patient Visual Analytics**	Patient Timeline	Single patient view
	Event Summary	A simplified summary of events
**Treatment-associated Analytics**	Sankey Visualization	Tracking changes in lines-of-treatment
	Heatmap Visualization	Visualizing the treatment frequency, duration, and combination.
	Swimmer's Plot	Cohort level visualization of a particular event
	Outlier Analysis	Identify extended treatment regimen
**Time-to-Event Analytics**	Swimmer's Plot	Visualization of the Time origin and Event time
	Survival Analysis	Kaplan Meier and Cox proportional hazard analysis.
**Cohort Overview**	Event Summary	A table of all events
	Swimmer's Plot	Cohort level visualization of all events
	Annotate Event Position	Integrate table
	Cohort Stratification	Patient Stratification

**Table 2:** Start and end point definitions.

	Start	End point
Overall Survival	From Diagnosis	Death or last follow-up/contact
Time-to-treatment-end	Treatment Start	Change in Treatment
		Treatment-associated toxicity
		Death
Real-World Progression Free Survival	Treatment Start	Change in Treatment-Line
		Progression/Metastasis
		Death

## Data Availability

Software code has been deposited inside the Zenodo repository: https://doi.org/10.5281/zenodo.16527381.
